# Structural Changes of Water in Carboxymethyl Cellulose Nanofiber Hydrogels during Vapor Swelling and Drying

**DOI:** 10.1021/acsomega.4c07831

**Published:** 2024-10-29

**Authors:** Yuta Takahara, Yusuke Beni, Yurina Sekine, Takuya Nankawa, Tomoko Ikeda-Fukazawa

**Affiliations:** † Department of Applied Chemistry, Meiji University, Kawasaki 214-8571, Kanagawa, Japan; ‡ Promotion Office, Japan Atomic Energy Agency (JAEA), Tokai 319-1195, Ibaraki, Japan

## Abstract

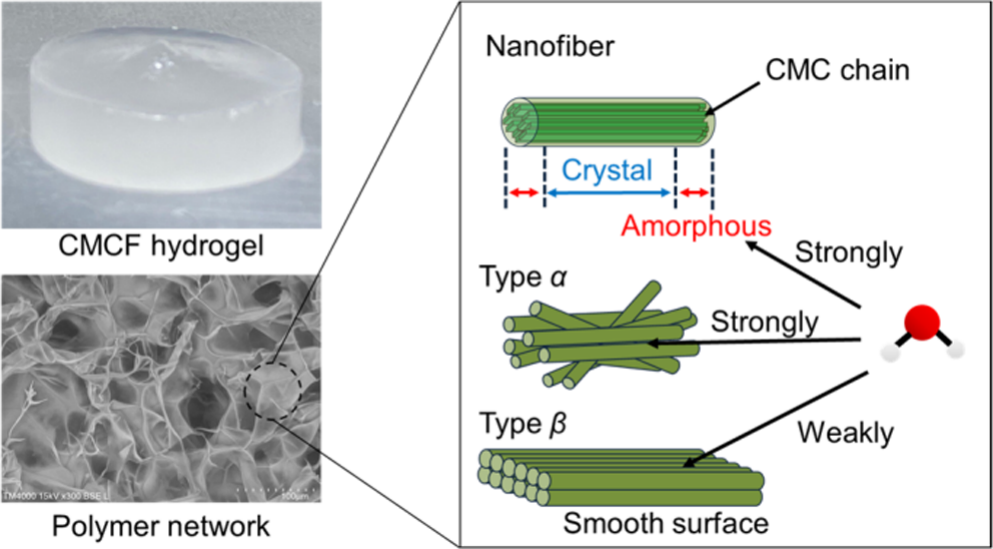

Carboxymethyl cellulose nanofiber (CMCF) forms mechanically strong hydrogels via freeze cross-linking. We investigated the vapor swelling and drying processes of the freeze cross-linked CMCF hydrogels using infrared spectroscopy and X-ray diffraction. From the shifts of the O–H and C=O stretching modes, the structural changes of water and carboxymethyl celluloses (CMC) were analyzed. The results show that two types of bound water exist in CMCF hydrogels due to a difference in hydrophilicity between the amorphous and crystalline regions of CMCF. Bound water adsorbed on the amorphous region forms a strong hydrogen bond with dangling O–H or C=O bonds of CMC, whereas that adsorbed on the crystalline region has a weak hydrogen bond with the localized hydrophilic groups on the hydrophobic surface. Due to the difference in the hydrogen bonding strength of the two types of bound water, the vapor swelling process of water in CMCF hydrogels is classified into four stages. For the drying process, the residual water, which formed a strong hydrogen bond with the hydrophilic groups of the CMC, has effects on the CMCF structure. The present result suggests that the adsorption and desorption of water are important factors governing the physical and chemical properties of the CMCF hydrogels.

## 1 Introduction

**Figure 1:**
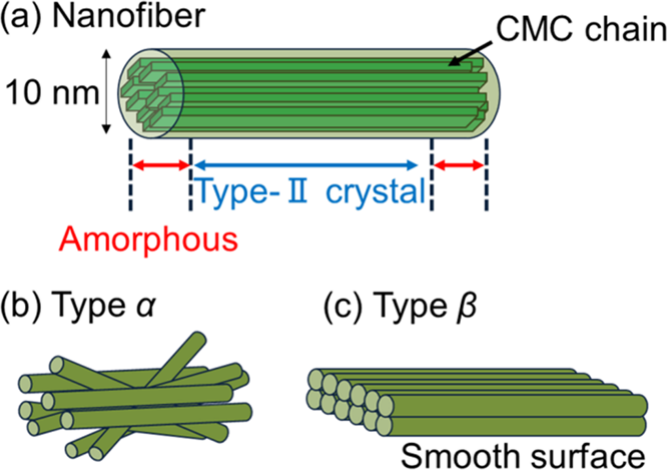
Schematics of structures of (a) CMCF fibril, (b) type α, and (c) type β fibril aggregations.

Cellulose is an abundant natural material on the earth. For the effective use of cellulose, many studies have been performed to investigate the properties and structures of cellulose using various methods.^1−8^ Crystalline and amorphous states coexist in cellulose material. Cellulose crystal can be classified into seven types, namely, I_α_, I_β_, II, III_I_, III_II_, IV, and IV_II_. The crystallographic structures of type-I_α_ and I_β_ crystals are triclinic in space group *P*1 and monoclinic in space group *P*2_1_, respectively.^9−11^ In the type-I_β_ crystal, cellulose chains are parallelly arranged along [001] axes on {200} planes. Although the crystallographic structure of the type-II crystal is the same as that of type-I_β_ (i.e., monoclinic in space group *P*2_1_), cellulose chains arrange in an antiparallel orientation along [001] axes on {020} planes in the type-II crystal.^12^ Due to the difference in the arrangements of cellulose chains, the formation sites of hydrogen bonds between intra- and interchains depend on the type of crystal. The type-I crystal, which is a mixture of type-I_α_ and I_β_, is an abundant phase in nature and transforms into type-II structure with swelling under a wet condition at room temperature.^13^ Furthermore, the transformation from type-I to II has been found in nanocellulose hydrogels.^14^ The hydrophilicity of cellulose in solid state is attributed to the coexisting amorphous state because hydroxyl groups without hydrogen bonds exist in the amorphous region.^15^ The amorphous region exists in the ends of a CMC fibril (Figure 1 (a)) and in the boundaries between the crystalline phases.

The structures and properties of absorbed water in cellulosic materials have been studied using various methods such as small-angle X-ray scattering (SAXS), X-ray diffraction (XRD), infrared (IR) spectroscopy, near-infrared (NIR) spectroscopy, differential scanning calorimetry (DSC), and molecular dynamics (MD) simulation.^16−26^ Nakamura et al.^23^ categorized the water adsorbed on cellulose materials into three types. In general, water in hydrogels and aqueous solutions has been classified into three types: free water, intermediate water, and bound water.^27^ Free water refers to water molecules behaving as those in bulk water, and the interactions with polymer chains are negligible. Bound water is usually associated with water molecules that form hydrogen bonds with polar polymers or strongly interact with polymer chains. The polar functional groups on the hydrophilic polymer chains can capture water molecules through hydrogen bonds.^28^ Intermediate water exists between the free water and bound water and interacts weakly with the polymer chains. Intermediate water is further classified into two types: first and second intermediate waters.^29^ First intermediate water forms hydrogen bonds with bound water, whereas the second intermediate water is structured around the polymer chain by hydrophobic interactions. The four types of water exhibit different properties, such as solubility, diffusivity, and equilibrium vapor pressure.^30^

Nakamura et al.^23^ showed that the amount of bound water in cellulosic materials correlated with the relative volume of amorphous regions of celluloses. Lindh et al.^31^ further categorized the bound water of cellulose into two types: mobile and immobile bound waters. The mobile bound water adsorbs on the external surface of microfibril aggregates or at the boundaries between aggregates, whereas immobile bound water is accommodated between the microfibrils in aggregates.^31,32^ Adsorbed water around crystal region prevents the cellulose materials from aging due to the formation of hydrogen bonds with the hydroxyl groups on surface.^19,33^ In contrast, adsorbed water in amorphous region causes a decrease in crystallinity because of the distortion of the periodic structure.^34^ This distortion causes a plasticizing effect in the polymer matrix. The results suggest that an understanding of the structure and behavior of water coexisting with cellulose is essential for the application and further development of cellulose materials.

Owing to their biodegradability, environmental friendliness, and swelling capabilities, cellulose hydrogels have attracted interest as products for various industries.^35^ The gelation of cellulose occurs with the formation of intermolecular hydrogen bonds, covalent bonds, and ionic interaction.^36−39^ Kimura et al.^36^ showed that a strong cellulose hydrogel was formed via stepwise solvent exchange after the dissolution of cellulose in an ionic liquid solution. Suenaga and Osada^37^ proposed a gelation method for cellulose nanofibers (CNFs) without chemical modification using a hydrothermal treatment. They showed that the mechanical strength of the formed hydrogel increased owing to an increase in the physical network structure of the CNFs.

Recently, Sekine et al.^40^ have developed a freeze cross-linking method to form hydrogel of carboxymethyl cellulose nanofiber (CMCF). They showed that a mechanically strong hydrogel can be prepared by thawing a frozen CMCF sol after adding an aqueous solution of citric acid (CA).^40^Figure S1 shows the chemical structures of carboxymethyl cellulose (CMC) and CA. A three-dimensional network structure of CMCF is formed by the aggregation of nanofibers with exclusion from the water phase during the formation and growth of ice crystals. The weak network structure of CMCF is strengthened by the formation of hydrogen bonds with CA between their carboxy and hydroxy groups. The aggregates of CMCF exist as the cellulose type-II crystal and amorphous phase.^41^ Miura et al.^41^ analyzed the structural change of CMCF aggregations during the freeze cross-linking reaction and showed that the sheet structure of CMCF aggregations transformed from a fibrous surface (types α) to a smooth surface (types β). Figure 1 shows schematic illustrations of structures for CMCF aggregations of types α and β.

To investigate the effects of the CMCF structures on the structure and properties of water, we analyzed the structural changes of water and CMC in freeze cross-linked CMCF hydrogels during vapor swelling and drying using IR spectroscopy. The peaks of the bending mode of water, the C=O stretching modes of CMC and CA, and the O–H stretching modes of water and CMC were mainly used for the analyses of the IR spectra. In addition, the change in the crystallinity of CMCF during natural drying was investigated by XRD.

## 2 Experimental Section

### 2.1 Preparation of Hydrogels

A 2 wt % CMCF aqueous sol (Sugino Machine Co., Ltd., TFo-10002) and CA (FUJIFILM Wako Pure Chemical Co., Ltd., Japan) were used for sample preparation without any purification. Before being frozen, the CMCF sol (2.0 g) was poured into a sample cup and placed in an incubator (EYELA, LTI-700) at 274 K for 24 h to remove air bubbles. A resin mold with a diameter of 30 mm, an aluminum cap with a diameter of 20 mm, and an aluminum sample plate were used as sample cups to prepare samples for scanning electron microscopy (SEM) observation, XRD measurements, and IR measurements, respectively. The CMCF sol was frozen in a freezer (SANYO, SR-111) at 248 K for 24 h. Then, a 1 mol L^–1^ CA aqueous solution (2.0 mL) was added to the frozen CMCF sol. The mixture was placed in an incubator at 274 K for 24 h, and then the mixture was soaked in distilled water for 48 h to remove the unreacted CA. The formed gels were freeze-dried using a diaphragm pump (KNF, N920 KT.29.18) and a cooling trap (EYELA, UNI TRAP UT-1000).

For the IR and XRD measurements, the freeze-dried samples were swollen in an atmosphere with a controlled water vapor content to equilibrium vapor pressure (∼3 kPa) at room temperature for more than 24 h before the measurements. The water content (WC) of the swollen samples was determined as follows


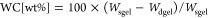
1where *W*_sgel_ and *W*_dgel_ are the weights of the swollen and freeze-dried hydrogels, respectively. For the natural drying, the samples were stored in a desiccator at room temperature (296 ± 3 K) and a humidity of 50 ± 10%.

### 2.2 SEM Observations

The network structures of the freeze-dried hydrogel samples were observed using SEM (Hitachi High-Tech, Miniscope TM4000PlusII) at an accelerating voltage of 15 kV.

### 2.3 XRD Measurements

XRD measurements were performed on freeze-dried and swollen samples with a water content of 2.0 wt % at room temperature. The water content (2.0 wt %) of the swollen sample was determined from the weights of the sample before and after the XRD measurement. The value was almost constant during the measurement. An X-ray diffractometer (Rigaku, Ultima IV) with Cu Kα radiation of 0.15418 nm in wavelength was used for the measurements. The diffraction data were collected with a step of 0.01° in the 2θ range of 5–90°.

The XRD pattern in the range of 5–50° was decomposed into seven modes by fitting the data. Lorentzian functions were used for the three peaks of crystalline cellulose and two peaks of amorphous cellulose, and Gaussian functions were used for the two peaks of water.^29,42−46^ The crystallinity index, Cr.I., was estimated using the following equation^44^
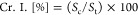
2where *S*_c_ and *S*_t_ are the integrated intensities of the two peaks assigned to crystalline cellulose and five peaks of the amorphous and crystalline celluloses, respectively.^44^

### 2.4 Infrared Spectroscopic Measurements

The IR spectra were measured at room temperature during the natural drying (i.e., air drying by keeping the sample in the atmosphere) of the vapor swelled samples. A Fourier transform IR spectrometer (JASCO, FTIR-4100) with an attenuated total reflection (ATR) unit (JASCO, ATR PRO450-S) was used for the measurements. In the ATR unit, the samples were pressed onto a ZnSe crystal by using a flexible metal head. The spectrum was obtained by integrating 32 scans in the wavenumber range of 650–4000 cm^–1^ at a resolution of 2 cm^–1^. The IR measurements were performed every 5 min: 2 min for measurement under the press with an interval of 3 min for drying without pressing.

The water content of the hydrogels during the IR measurements was determined using the integrated intensity ratio of the bending mode of water to the C=O stretching mode of CMC, *I*_δ (HOH)_/*I*_ν (C=O)_. Using the ratio of integrated intensities (ROI), the mass ratio of water to CMCF, *W*_water_/*W*_CMCF_, is given by
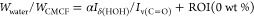
3where *W*_water_ and *W*_CMCF_ are the weights of water and CMCF, respectively. *I*_δ (HOH)_ and *I*_ν (C=O)_ are the integrated intensities of the bending mode of water and the C=O stretching mode, respectively. α and ROI (0 wt %) are constants determined from the IR spectra of the samples during vapor swelling. Figure S2 shows the relationship between *I*_δ (HOH)_/*I*_ν (C=O)_ and *W*_water_/*W*_CMCF_ during vapor swelling. The gradient α and intercept ROI (0 wt %) of the calibration line (dotted line in Figure S2) are 2.559 and 0.5083, respectively. The water content of the samples during natural drying was estimated by using the calibration line. The *W*_water_/*W*_CMCF_ value can be converted to water content as (*W*_water_/*W*_CMCF_)/(1 + (*W*_water_/*W*_CMCF_)).

To analyze the wavenumbers of the respective modes, analyses were performed for the IR spectra without an ATR correction. The ATR correction was not applied, because the changes in the spectra measured using the same method were analyzed. In the region of 1500–1800 cm^–1^, the spectra were decomposed into two Gaussian functions: the bending mode of water and the C=O stretching mode of CMC.^40^ The intensities of all of the spectra were normalized by the intensity of the C–H stretching band. In the region of 3000–3800 cm^–1^, the O–H stretching modes of water and CMC coexisted. The O–H stretching band of water in the region of 3000–3800 cm^–1^ was analyzed using a three-state model of water: (i) immobile “network” water, (ii), mobile “network” water, and (iii) less-structured “multimer” water.

## 3 Results and Discussion

### 3.1 Structure of CMCF Networks

**Figure 2:**
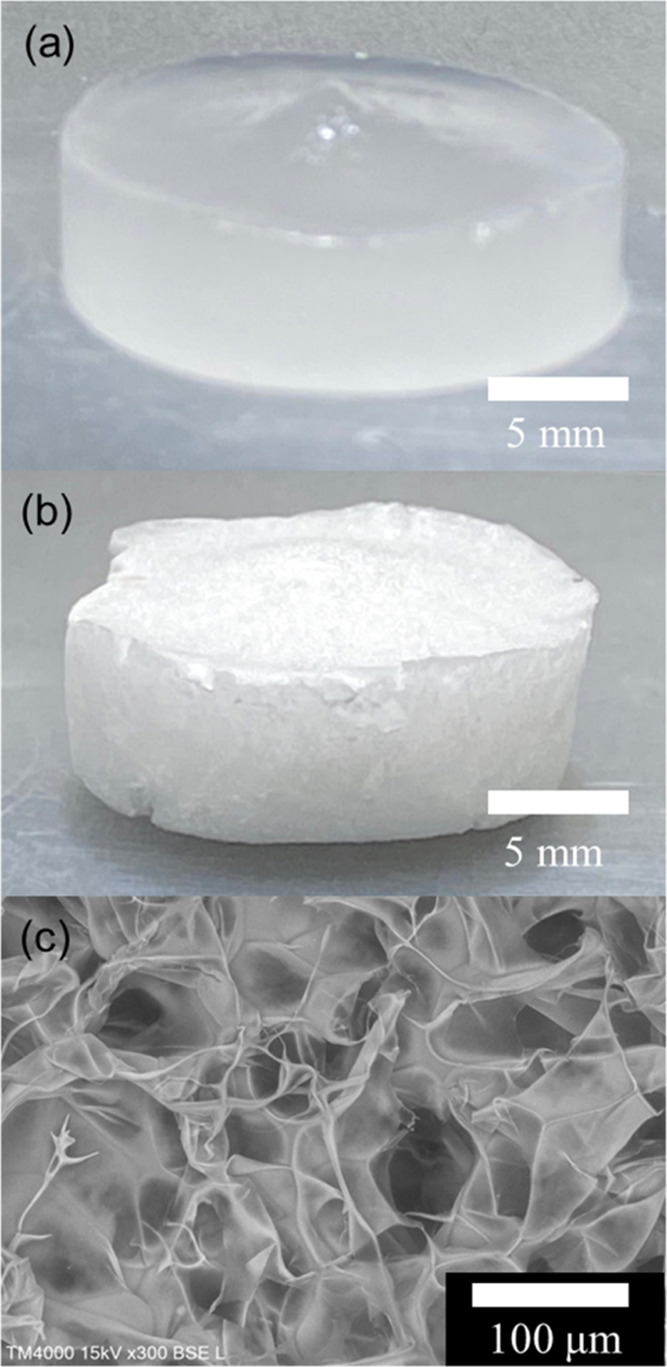
Photographs of a CMCF hydrogel (a) before and (b) after freeze-drying. (c) SEM image of a freeze-dried CMCF hydrogel.

Figure 2(a,b) shows photographs of a CMCF hydrogel before and after freeze-drying, respectively. The linear shrinkage rate with the freeze-drying is approximately 80.6%. The shrinkage rate was determined by the ratio of the thickness of the cylindrical sample to that before the drying. Thus, the freeze-dried sample approximately maintains the network structures of CMCF in its swollen state. Figure 2(c) shows the SEM image of the freeze-dried CMCF hydrogel. Micropores of approximately 80 μm in diameter and sheet-like structures with a smooth surface are observed in the SEM image. The CMCF hydrogel has a high water content owing to the micropores.^40^

**Figure 3:**
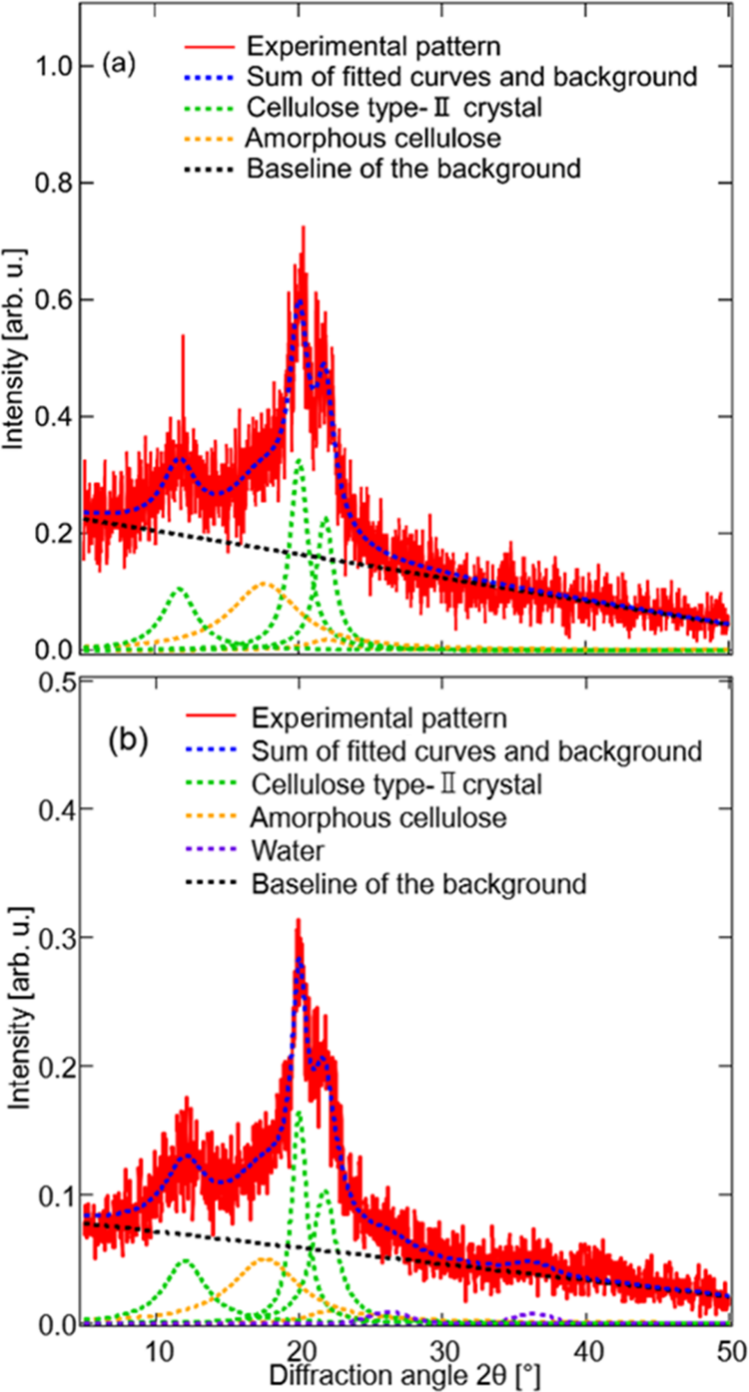
XRD profiles of (a) the freeze-dried CMCF hydrogel and (b) the naturally dried CMCF hydrogel after vapor swelling. Red lines show the experimental patterns. Black dotted lines are the baseline of the background. Green, orange, purple, and blue dotted lines are the fitting curves of the cellulose type-II crystal, amorphous cellulose, water, and sum of the fitted curves and background, respectively.

The Cr.I. value of the freeze-dried CMCF hydrogel was estimated from the XRD profile. Figure 3(a) shows the XRD profile of the freeze-dried CMCF hydrogel. Three characteristic peaks, denoted by the green dotted lines, are observed at 11.98, 19.98, and 21.76°. These peaks are assigned to (1̅10), (110), and (020) planes of the type-II cellulose crystal, respectively.^42−46^ The broad peaks (orange dotted lines) are assigned to the halos of the amorphous region.^43^ Using the integrated intensities of these five peaks and eq 2, the Cr.I. value of the freeze-dried CMCF hydrogel was determined to be 57.08%. This value is consistent with the reported values for mercerized and regenerated celluloses (61.02^44^ and 65–74%^47^).

The low Cr.I. value (i.e., 57.08%) indicates that the type-II crystal and amorphous phase coexist in the CMCF hydrogel. The amorphous cellulose exists in the ends of a CMC fibril, as shown in Figure 1(a). The ends of the fibril have an amorphous structure, although the CMC chains periodically arrange to form the type-II crystal in the inner part of the fibril.

### 3.2 Structural Changes of Water with Swelling

As described above, the crystal and amorphous regions coexist in the CMCF hydrogel. Due to the difference in the hydrophilicity between the regions, the adsorption site of water in CMCF hydrogel during swelling is expected to depend on water content. In the beginning of the swelling process (i.e., at lower water contents), water is preferentially absorbed around the amorphous region and at the boundaries between aggregates because of the formation of hydrogen bonds with dangling O–H or C=O bonds.^21^ Subsequently, the water adsorption around the crystalline region, which has a hydrophobic plane ((110) surface), occurs via the formation of weak hydrogen bonds with the side chains on the surface.^48^ To investigate the effects of cellulose structures on water structures, the IR spectra of CMCF hydrogels were analyzed during vapor swelling.

**Figure 4:**
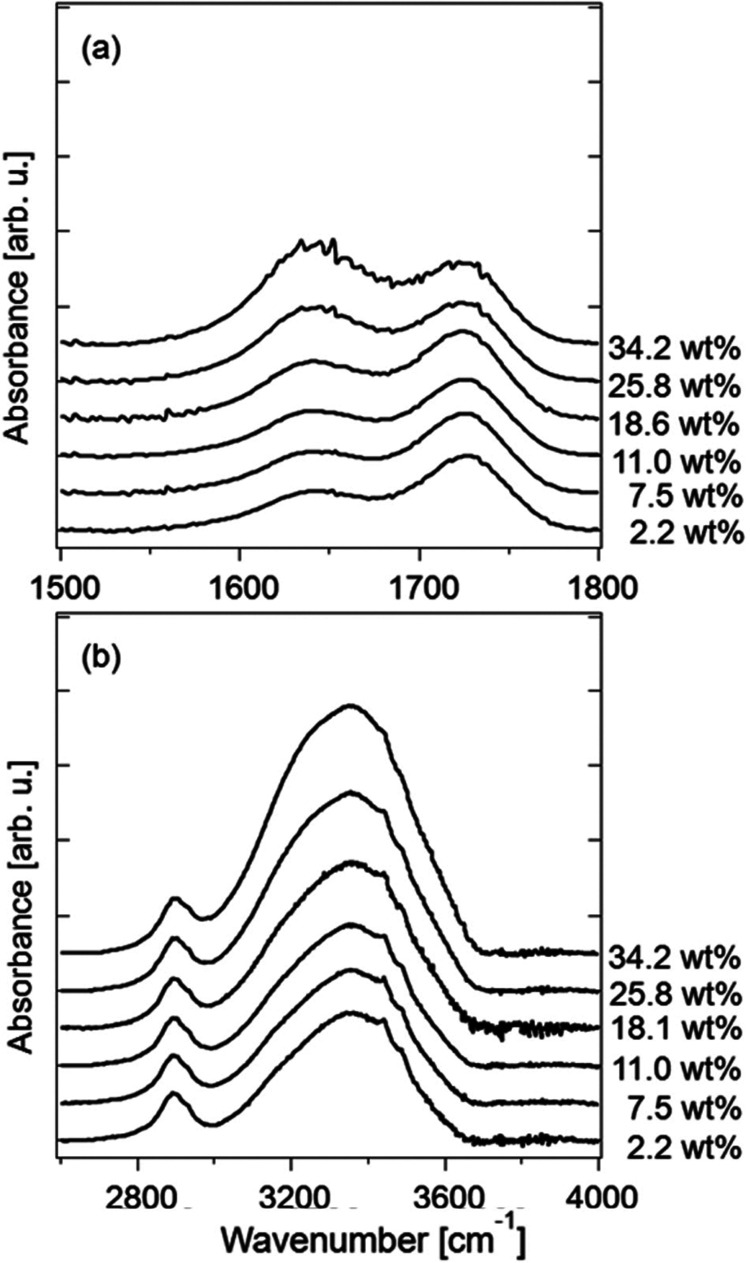
IR spectra in the regions of (a) H–O–H bending and (b) the O–H stretching modes of a CMCF hydrogel during the vapor swelling process.

Figure 4(a,b) shows the IR spectra of the CMCF hydrogel during vapor swelling in the regions 1500–1800 and 2600–4000 cm^–1^ after a baseline correction. A linear baseline was applied to each spectrum so that the values at 1500, 1800, 2600, and 4000 cm^–1^ become zero. The broad band observed in 3000–3800 cm^–1^ is assigned to the overlapping peaks of the O–H stretching modes of CMC and water. The intensity of the band increases as the water content increases because of the large contribution of water to the band. The peaks at around 2900 cm^–1^ are attributed to the C–H stretching modes of the CH and CH_2_ units in CMC and CA (Figure S1). The peaks at 1640 and 1723 cm^–1^ are assigned to the bending mode of water and the C=O stretching mode of CMC, respectively.^40^ The relative intensity of the bending mode of water increases as the water content increases. Figure S2 shows the intensities ratio of these two modes as a function of the mass ratio of water to CMCF. The calibration line shown in Figure S2 was used to estimate the water content during natural drying.

As shown in Figure 4(b), the O–H stretching modes of CMC and water coexist in the region of 3000–3800 cm^–1^. The sharp peaks observed at 3442 and 3488 cm^–1^ are assigned to the O–H stretching modes of the O3–H unit in the CMC. As shown in Figure S3, the oxygen in the CMC is classified into six sites (O, O2, O3, O5, O6, and O in CH_2_COOH). The hydrogen of the O3–H unit forms hydrogen bonds with O5 or O6 in the type-II cellulose crystal.^2,49,50^ In addition to the O–H stretching modes of CMC, there are several modes of water in the region.^51,52^Figure S4 shows a typical decomposed spectrum of the CMCF hydrogel using five Gaussian functions. The peaks are observed at 3050, 3230, 3400, 3540, and 3625 cm^–1^. The peaks at 3230 and 3400 cm^–1^ are assigned to the O–H stretching modes with strong hydrogen bonds, and the peaks at 3540 and 3625 cm^–1^ correspond to the O–H stretching modes with weak or less hydrogen bond.^51^ The peak at 3050 cm^–1^ is assigned to the Fermi resonance between the overtone of the bending mode and the O–H stretching mode of water with strong hydrogen bond.^51^

We attempted to analyze the spectra using a three-state model of water: (i) immobile “network” water, (ii) mobile “network” water, and (iii) less-structured “multimer” water. In addition to the two-state model of water (i.e., “network” and less-structured “multimer” water) proposed by Binder,^53^ we attempted to classify “network” water into two types. The immobile “network” water forms strong hydrogen bonds with the dangling O–H or C=O bonds at the boundaries between CMCF aggregates, whereas mobile “network” water forms weak hydrogen bonds with the side chains on the surface of the crystalline region of CMCF.^21,48^ Binder^53^ assigned the peak with maximum intensity near 3400 cm^–1^ and shoulder near 3250 cm^–1^ to the antisymmetric and symmetric modes of the O–H stretching of “network” water. The shoulder near 3600 cm^–1^ was assigned to the O–H stretching of less-structured “multimer” water. We assigned the shoulder near 3250 cm^–1^ to the mobile “network” water with weak hydrogen bonds, and shoulder near 3150 cm^–1^ to the immobile “network” water with strong hydrogen bonds.

The structural changes of water were analyzed by using the wavenumbers and absorbance ratios. The integrated absorbance over a spectral range of ±20 cm^–1^ for the selected position in the spectrum was used for the analyses of the absorbance ratio.^53^ The integrated absorbance for the position at the wavenumber of *i* cm^–1^ was defined as *A*_*i*_ = ∫*A*(*ν*) d*ν*/Δ*ν*, where *i* = 3600, 3250, and 3150.^53^ Using the *A*_*i*_ values, the absorbance ratios *R*_3600/3250_ (= *A*_3600_/*A*_3250_) and *R*_3150/3250_ (=*A*_3150_/*A*_3250_) were analyzed.

**Figure 5:**
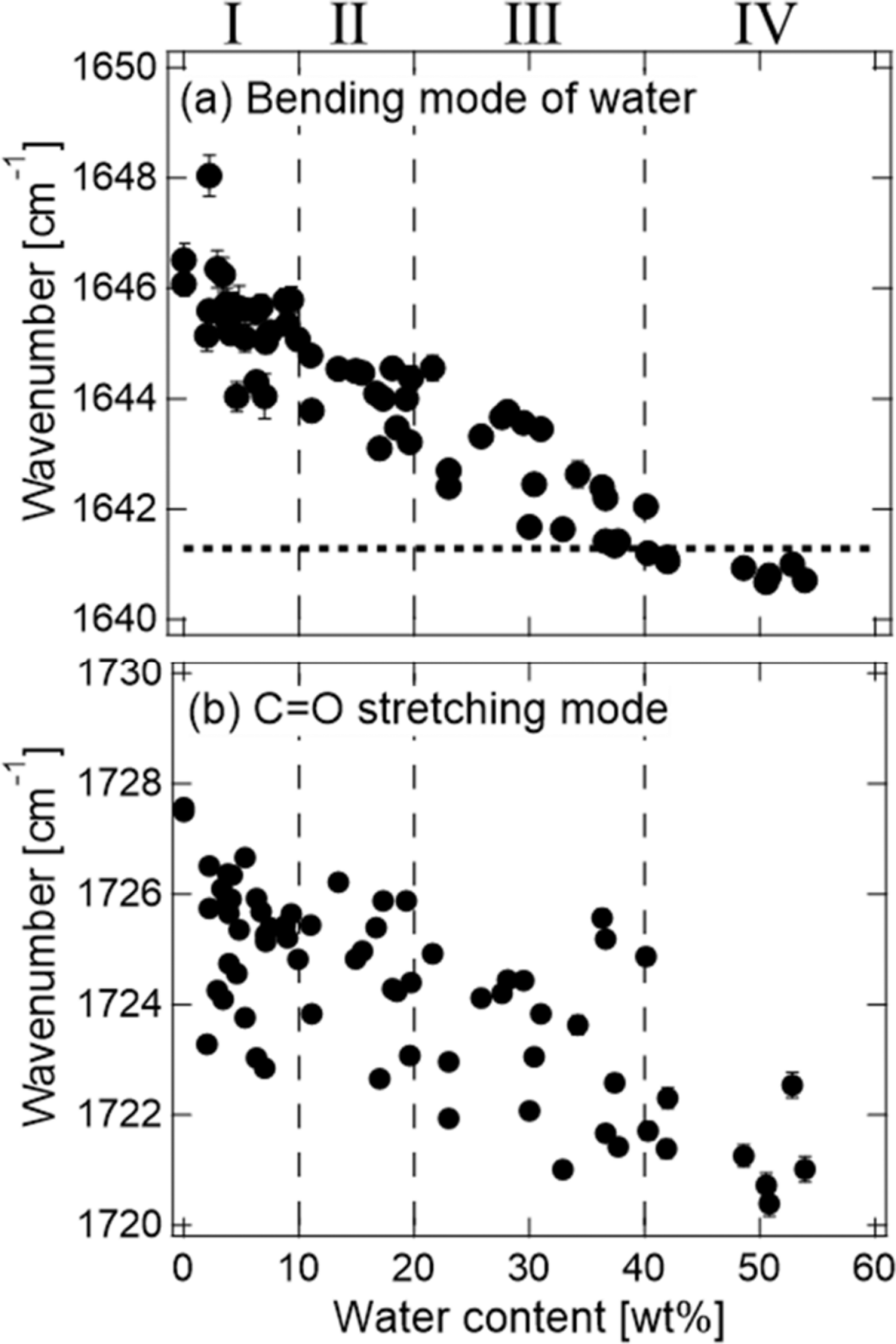
Dependences on water content of wavenumbers of the (a) bending mode of water and (b) C=O stretching mode of CMC and CA during the vapor swelling process. The dotted line in (a) shows the wavenumber of pure water. The dashed lines denote the boundaries between the vapor swelling stages.

**Figure 6:**
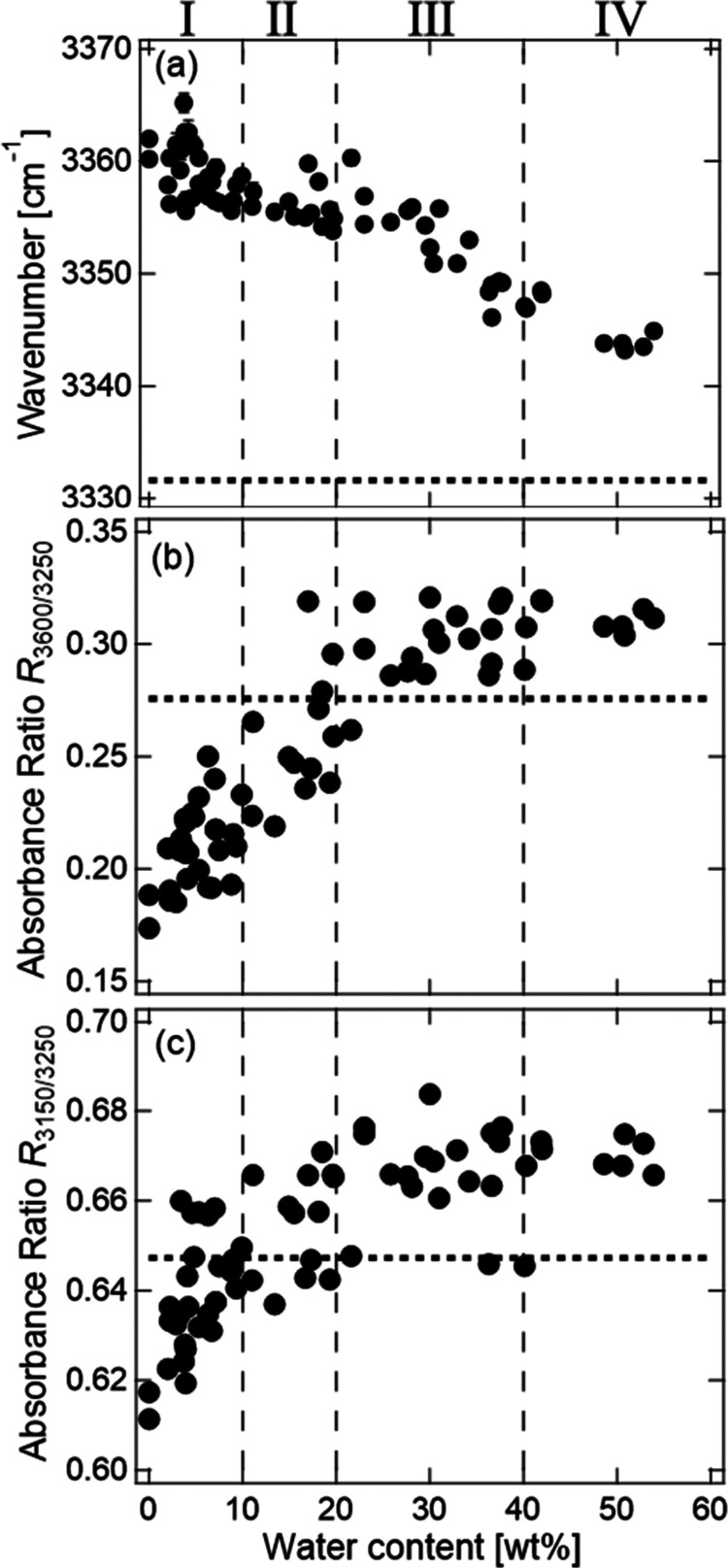
Dependence on water content of (a) wavenumber of peak with maximum intensity of the O–H band, absorbance ratios at (b) 3600 and 3250 cm^–1^, and (c) 3150 and 3250 cm^–1^. The dotted lines indicate the values of pure water. The broken lines show the boundaries between the vapor swelling stages.

Figures 5 and 6(a) show the change in the wavenumbers of the bending mode of water, the C=O stretching mode of CMC, and the peak with maximum intensity in the O–H stretching region. The bending mode of water, which exists independently of the peaks of CMC and CA, is used as a measure of water content in the hydrogel. The wavenumber of the C=O stretching mode decreases as the water content increases. This result suggests that the formation of hydrogen bonds of water with the C=O bonds in CMC proceeds with swelling. Figure 6(b) shows the absorbance ratio of the O–H band at 3600 and 3250 cm^–1^, which can be a measure of the number ratio of “network” water and less-structured “multimer” water, as a function of water content. Figure 6(c) shows the ratio at 3150 and 3250 cm^–1^ as a measure of the immobile and mobile “network” water.

Based on the changing rates of the wavenumbers and absorbance ratios of Figures 5 and 6, the vapor swelling process is classified into four stages: 0–10 wt % (stage-I), 10–20 wt % (stage-II), 20–40 wt % (stage-III), and >40 wt % (stage-IV). The dashed lines in Figures 5 and 6 indicate the boundaries between the stages. In stage-I, the wavenumber of the peak position of the O–H band significantly decreases, and the absorbance ratios *R*_3600/3250_ and *R*_3150/3250_ increase as the water content increases. In stage-II, the wavenumber of the peak position of the O–H band continues to decrease and *R*_3600/3250_ continues to increase, while *R*_3150/3250_ scatters around a constant value. In stage-III, the wavenumber of the peak position of the O–H stretching band continues to decrease, whereas *R*_3600/3250_ and *R*_3150/3250_ are constants. In stage-IV, the wavenumber of the peak position of the O–H band also becomes a constant.

The changes in the wavenumber and absorbance ratio are attributed to the change in the adsorption site of water in the CMCF hydrogel during swelling. In stage-I, *R*_3600/3250_ and *R*_3150/3250_ increase as the water content increases. This indicates that the absorbed water in the beginning of stage-I is the immobile “network” water, that is, the bound water around the hydrophilic groups of CMC in the amorphous region and a boundary between aggregates. The higher wavenumber of the peak position than that of pure water suggests that the mode is assigned to the bound water, which forms a weak hydrogen bond with hydrophilic groups on the hydrophobic region.

**Figure 7:**
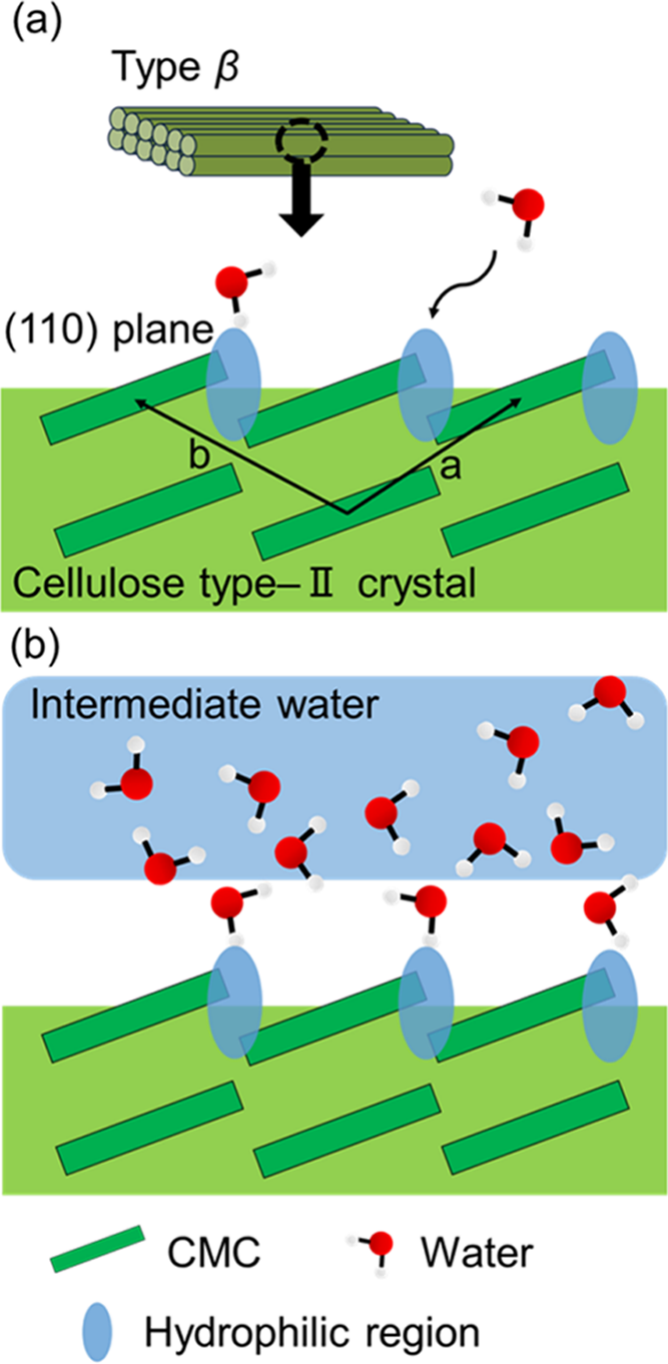
Schematic illustrations of adsorbed water on the surface of cellulose type-II crystal in (a) stages-I and II and (b) stages-III and IV.

In stage-II, the continuation of increase in *R*_3600/3250_ indicates that newly absorbed water molecules form hydrogen bonds with the bound water. In contrast, the constant *R*_3150/3250_ suggests that there is no difference in the formation rate of the mobile and immobile water molecules in this stage. In the end of stage-II, *R*_3600/3250_ approaches a constant value. This suggests that the adsorption of water on the hydrophobic crystalline surface begins in this stage. The mode near 3600 cm^–1^ corresponds to the adsorbed water on the surface of the sheet-like structure of cellulose type-II crystal. The hydrophilic and hydrophobic regions coexist periodically on the (110) plane of cellulose type-II crystal.^41^ Water molecules form hydrogen bonds with the O–H and C=O groups of side chain of CMC at the crystal surface (as shown in Figure 7(a)).^54^ The strength of hydrogen bonds of adsorbed water is weaker than that of pure water because the distance between the adsorbed water molecules is larger. The distance between adsorbed water molecules on the surface is governed by the distance between the hydrophobic regions.^55^

In stage-III, the wavenumbers of the O–H stretching mode continue to decrease, although *R*_3600/3250_ and *R*_3150/3250_ become constant. This result indicates that the intermediate water forms on the crystalline surface (Figure 7(b)) in stage-III. In stage-IV, most of newly adsorbed water exists as free water. The present results indicate that the water preferentially absorbs around the amorphous region and a boundary between aggregates in stage-I and absorbs on the crystalline surface in stages-II and III.

### 3.3 Structural Changes of Water with Drying

**Figure 8:**
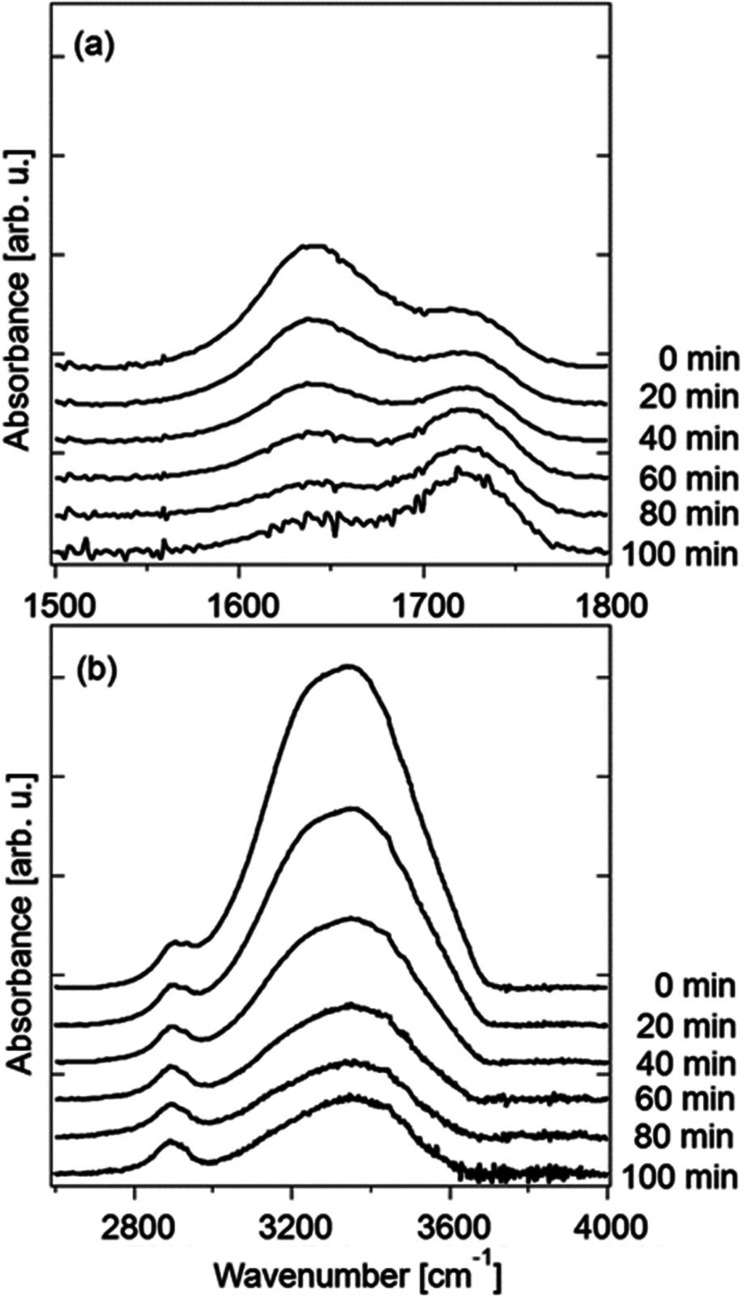
IR spectra in regions of (a) H–O–H bending and (b) the O–H stretching modes of the CMCF hydrogel during the drying process.

**Figure 9:**
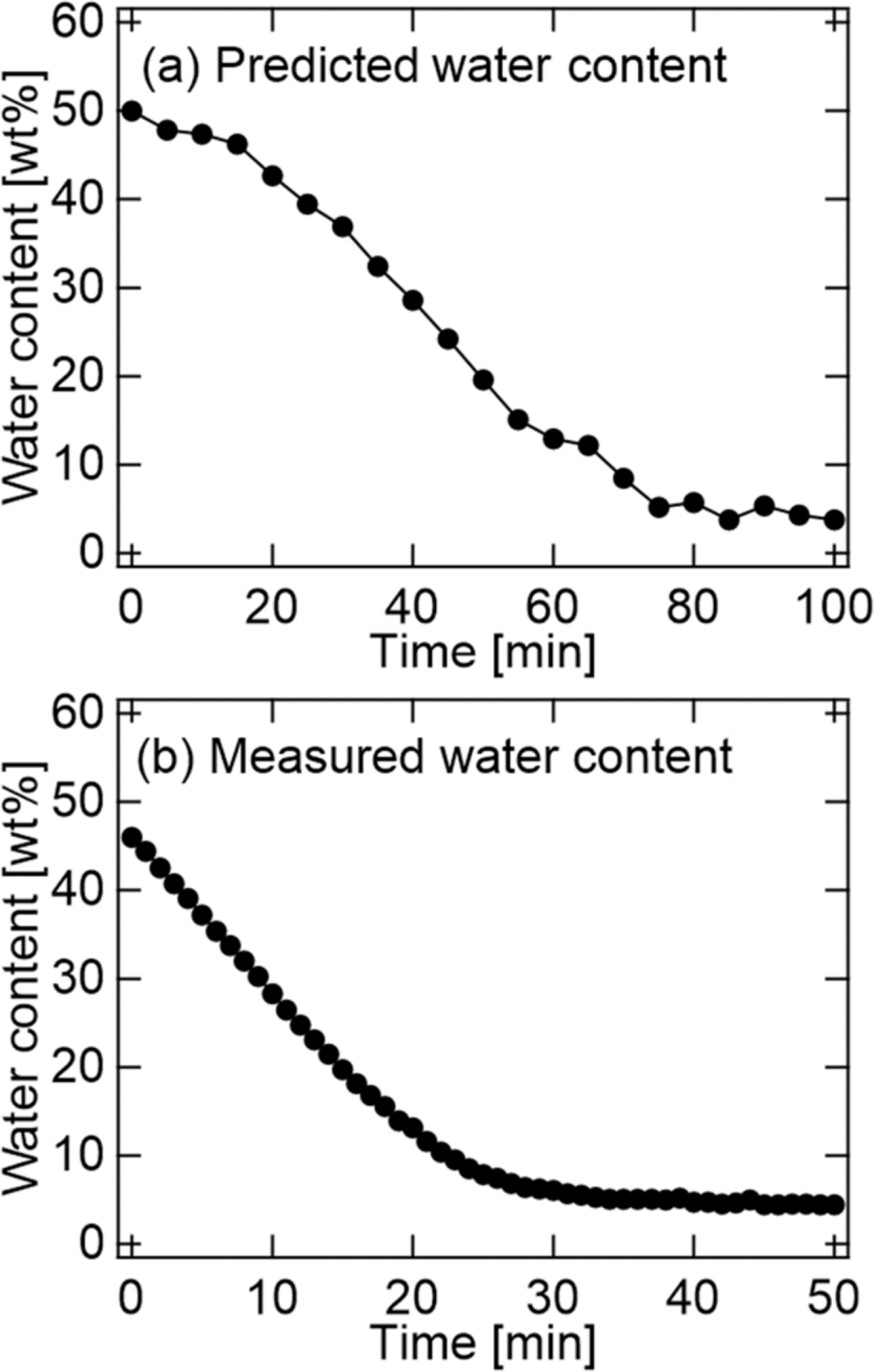
Temporal variations of (a) estimated and (b) measured water contents of the CMCF hydrogel.

Figure 8 shows the temporal variation of the IR spectra of the CMCF hydrogel during the drying process after vapor swelling to be 51.0 wt %. To estimate the water content using the IR spectra, we used eq 3 obtained from the calibration line shown in Figure S2. Figure 9 shows the estimates of water content from the IR spectra using eq 3 and those from the measured weights using eq 1. To investigate the influence of pressing on the sample during IR measurements, we measured the drying rate of the sample without pressing during natural drying (Figure 9(b)). As described above, the IR measurements were performed every 5 min (i.e., 2 min for the IR measurement under pressing and an interval of 3 min for drying without pressing). During the IR measurements under the press, the water content remained almost constant. Considering the interval during the IR measurements, no difference in the trend of the decreasing rate of the water content was observed for samples with and without pressing (Figure 9(a,b)). The water content obtained from the measured weights using an electronic balance before and after the IR measurement was 51.0 and 6.9 wt %, respectively.

**Figure 10:**
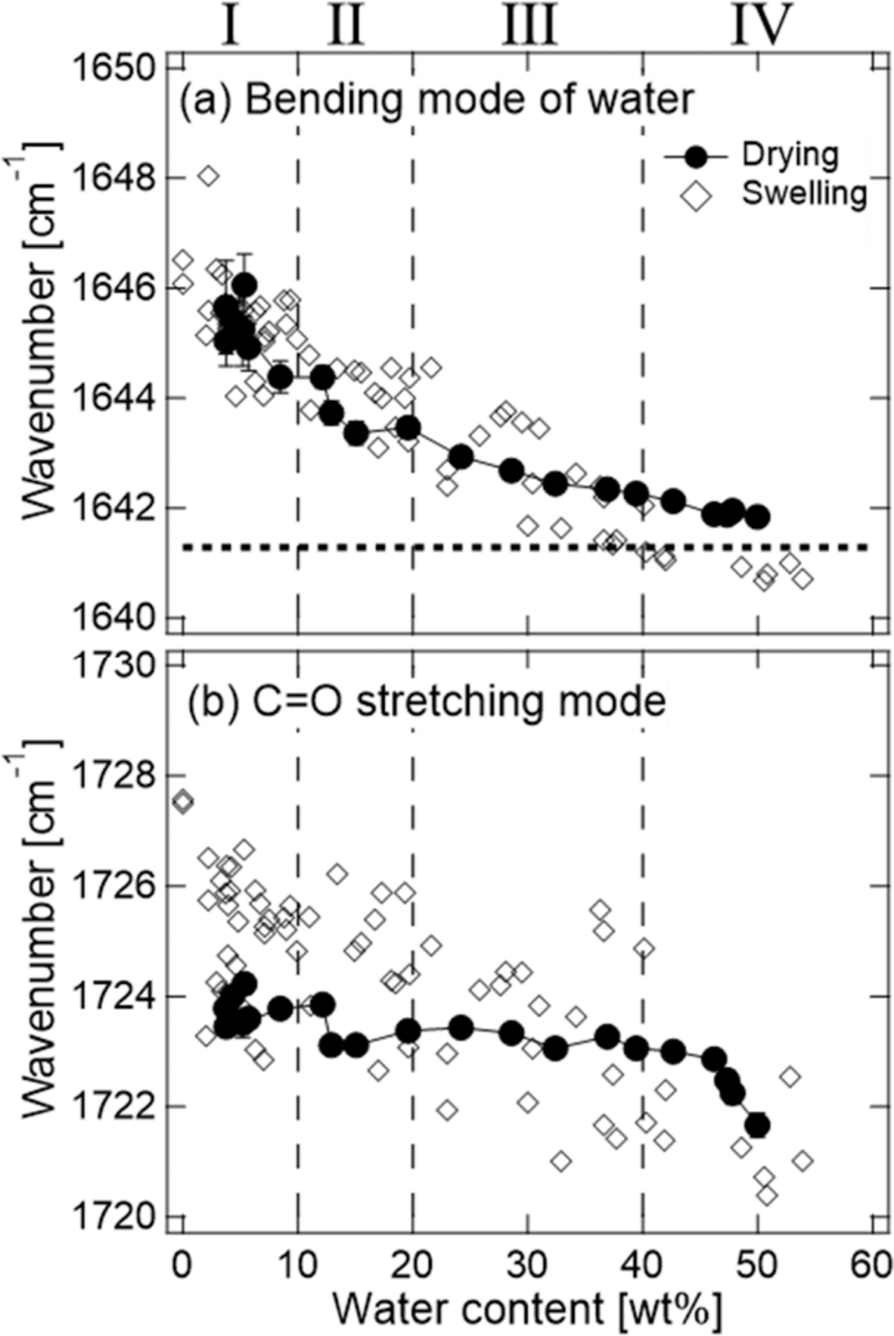
Dependences on water content of wavenumbers of the (a) bending mode of water and (b) C=O stretching mode of CMC and CA during the drying process (solid circles). The open diamonds are the data of the vapor swelling process. The dotted line in (a) shows the wavenumber of pure water. The dashed lines denote the boundaries between the vapor swelling stages.

**Figure 11:**
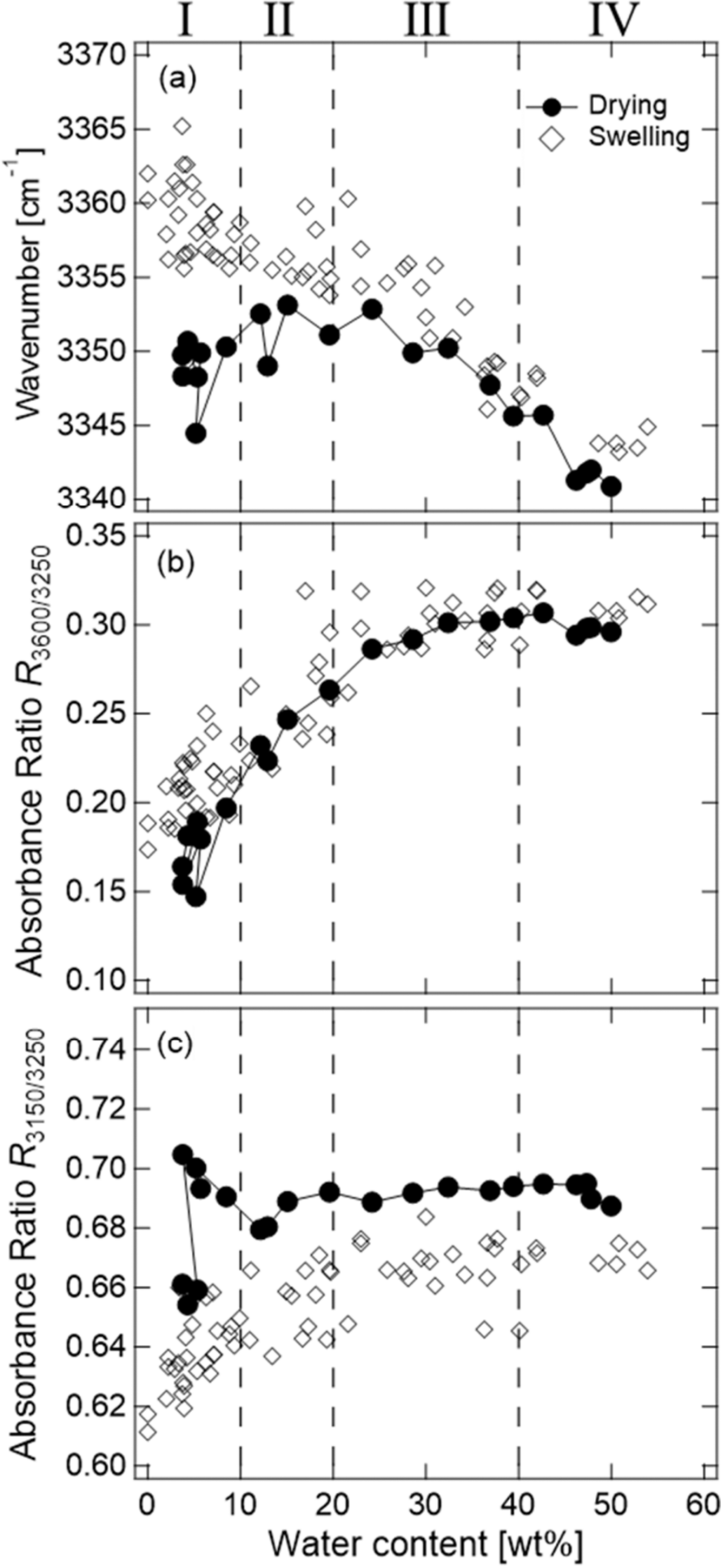
Dependence on water content of (a) wavenumber of peak with maximum intensity of the O–H band, absorbance ratios at (b) 3600 and 3250 cm^–1^, and (c) 3150 and 3250 cm^–1^ during the drying process (solid circles). The open diamonds are the data of the vapor swelling process. The dashed lines denote the boundaries between the vapor swelling stages.

As shown in Figure 8, the intensities of the O–H stretching and bending modes of water decrease with drying. Figures 10 and 11 show the changes in the wavenumber of the bending mode, peak with maximum intensity of the O–H band, and *R*_3600/3250_ and *R*_3150/3250_ during the drying process. The wavenumbers and absorbance ratio during the drying (solid circles) are consistent with those during the swelling (i.e., open diamonds) in stages-III and IV, whereas the differences are observed in stages-I and II. This result suggests that the remaining water exists after the drying. As shown in Figure S5, the intensity in the low-wavenumber region of the O–H stretching mode for the naturally dried sample is slightly larger than that for the freeze-dried sample. Furthermore, the wavenumber of the C=O stretching mode for the drying process is lower than that of the swelling process in stage-I as shown in Figure 10(b). These results indicate that a residual water, which forms hydrogen bonds with the C=O bond, exists in stages-I and II. This confirms that water with strong hydrogen bonds in the amorphous region and aggregation boundary remains without dehydration.

Figure 3(b) shows the XRD profile of the CMCF hydrogel with a water content of 2 wt % after natural drying from a vapor swelled state. The weak peaks observed at 26.31 ± 0.22 and 36.32 ± 0.21° are probably attributed to bound water because these values are closest to those of pure water (27.87 and 39.77°^56^). Using a Cu Kα wavelength of 0.15418 nm, the 2θ values of 26.31 ± 0.22 and 36.32 ± 0.21° are converted into *d*-spacing values of 0.339 ± 0.003 and 0.247 ± 0.001 nm, respectively. These peaks are assigned to the distances between the oxygen atoms in the second- and first-neighbor water molecules, respectively. The values are slightly larger than the *d* values of pure water (0.32 and 0.23 nm). The results show that the residual water in the dried CMCF hydrogel forms a weak hydrogen bond compared with that in pure water.^56^ This is consistent with the IR result. The *R*_3150/3250_ values in stage-I during the drying are higher than those of the swelling process as shown in Figure 11(c).

### 3.4 Structural Change of CMCF

We also analyzed the structural changes in CMCF during the vapor swelling and drying processes using the characteristic peaks of type-II cellulose crystal at 3442 and 3488 cm^–1^ in the IR spectra.^2,49,50^ These peaks are assigned to the O–H stretching modes of the O3–H (Figure S3). The difference in the wavenumbers of the two peaks is caused by the conformational differences in the side chains (gt or tg).^12,49,50,57^ The O3–H unit forms hydrogen bonds with O5 and O6 within the sheet. The difference in the wavenumbers between the two modes suggests that the strength of the hydrogen bonds depends on the binding site. The peaks at 3442 and 3488 cm^–1^ are assigned to the hydrogen bonds of O3–H with hydrogen bonds with O5 and O6, respectively.^50^

**Figure 12:**
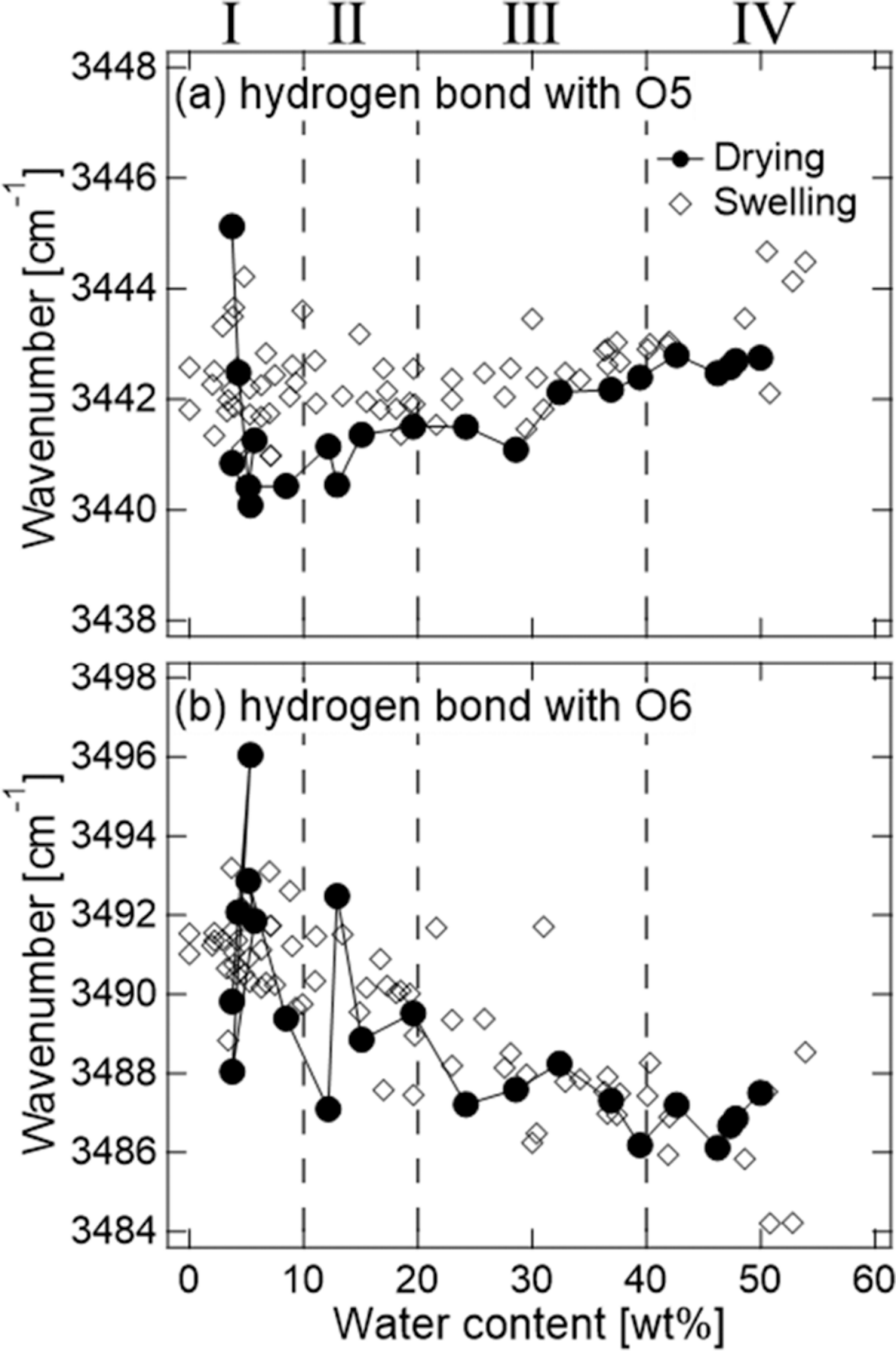
Dependence on the water content of wavenumbers of (a) O3–H, which mainly forms hydrogen bond with O5, mode, and (b) O3–H, which mainly forms hydrogen bond with O6, mode of CMCF during vapor swelling and drying processes. The open diamonds and solid circles show the data of the swelling and drying processes, respectively. The dashed lines denote the boundaries between the vapor swelling stages.

Figure 12 shows the changes in the wavenumbers of the two peaks of the type-II cellulose crystal during the vapor swelling and drying processes. In the swelling process, the wavenumber of the peak at 3488 cm^–1^ decreases as the water content increases, whereas that at 3442 cm^–1^ is almost constant. The result shows that the strength of the hydrogen bond with O6 increases with increasing water content. The strengths of the hydrogen bonds change due to the conformational changes of side chains of the CMC with the glass transition. Although the glass transition temperature of CMC is around 130 K, it increases as the water content decreases.^58^ In the case of hydrogels, a glass transition occurs with dehydration at room temperature.^16^ The mobility of the polymer network increases at temperatures above the glass transition point. Penetration of water molecules within the amorphous region^26^ and deformation of cellulose crystal with water adsorption^59^ are also possible causes of the conformational changes.

The structural change of the type-II cellulose crystal is also observed in the XRD profiles in Figure 2. The (1̅10) peak observed at 11.67° for the freeze-dried sample shifts to 12.04° after natural drying to be 2 wt % from a vapor swelling. These angles correspond to the *d*-spacings of 0.7583 and 0.7351 nm for the freeze-dried and naturally dried states with 2 wt %, respectively. This change suggests that the interval between the molecular layers decreases during the vapor swelling and drying processes. This structural change correlates with the differences in the conformations of the intermolecular hydrogen bonds.^50^ The strength of the intermolecular hydrogen bonds depends on the conformation of the side chain and the hydrogen bonds between O3–H and O6. These results suggest that adsorbed water affects the structures of cellulose in both the amorphous and crystalline regions.

## 4 Conclusions

To investigate the structural changes in water and their effects on the structure of CMCF during vapor swelling and drying, we measured the IR spectra and XRD patterns of the CMCF hydrogels. The results showed that there are two types of bound water in CMCF hydrogels: (i) bound water around the amorphous region and a boundary between aggregates, which forms a strong hydrogen bond with dangling O–H or C=O bonds of CMC, and (ii) bound water on the (110) surface of the type-II crystal, which forms weak hydrogen bond. Due to the difference in the hydrophilicity between the adsorption site of bound waters, the swelling process is classified into four stages: 0–10 wt % (stage-I), 10–20 wt % (stage-II), 20–40 wt % (stage-III), and >40 wt % (stage-IV). In stage-I, the absorbed water forms hydrogen bonds with the hydrophilic groups of the CMC and becomes bound water. In stage-II, formation of a water cluster around the bound water proceeds. In stages-III and IV, the adsorbed water mainly becomes intermediate and free waters, respectively. Furthermore, based on the changes in the wavenumbers of the two characteristic peaks of the type-II crystal, the conformational changes of the hydrogen bonds in the type-II crystal due to the water absorption were confirmed. The XRD profile showed that water adsorbed in the amorphous region has effects to change the structure of CMCF. The present results suggest that the adsorption and desorption of water are important factors governing the physical and chemical properties of CMCF hydrogels.

## Supporting Information Available

Chemical structures of CMC and CA, calibration line for estimating water content, oxygen sites in CMC, and IR spectra of CMCF hydrogels (PDF)
