# Supplementary material for: Structural Changes of Water in Carboxymethyl Cellulose Nanofiber Hydrogels during Vapor Swelling and Drying
Source: ACS Omega. 2024 Oct 29;9(45):45554–63. doi: 10.1021/acsomega.4c07831 (PMC11561596; doi:10.1021/acsomega.4c07831)
Supplement: Supplementary file 1 — ao4c07831_si_001.pdf [file ao4c07831_si_001.pdf]

Supporting Information

Structural Changes of Water in Carboxymethyl Cellulose Nanofiber Hydrogels during Vapor Swelling and Drying

Yuta Takahara,[1] Yusuke Beni,[1] Yurina Sekine,[2] Takuya Nankawa,[2] Tomoko Ikeda-Fukazawa[1,*]

[1] Department of Applied Chemistry, Meiji University, Kawasaki, Kanagawa, 214-8571, Japan.

[2] Promotion Office, Japan Atomic Energy Agency (JAEA), Tokai, Ibaraki 319-1195, Japan.

* To whom correspondence should be addresses (Tomoko Ikeda Fukazawa: fukazawa@meiji.ac.jp)

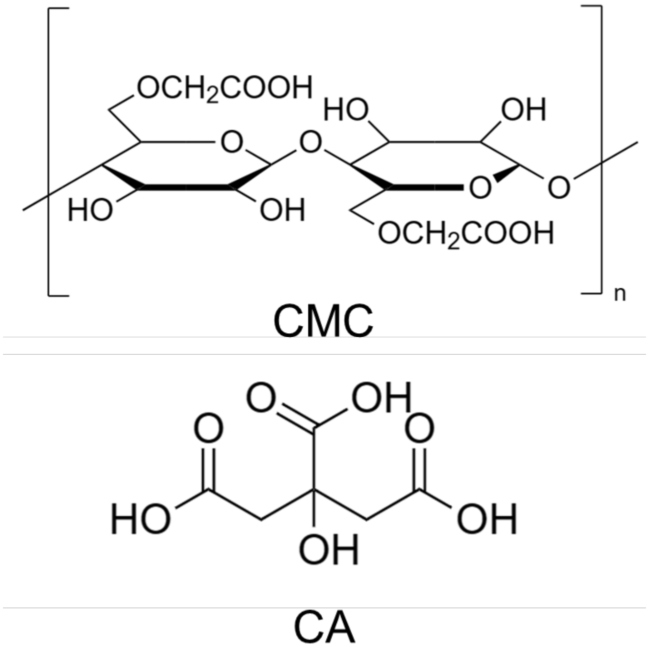

Figure S1. Chemical structures of CMC and CA.

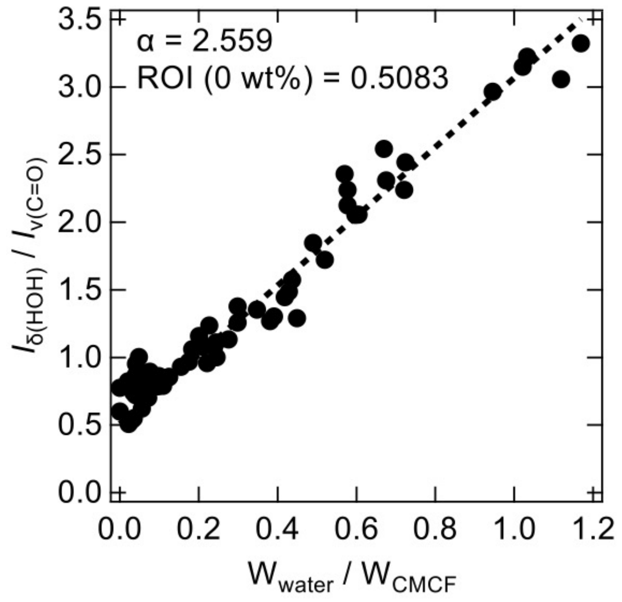

Figure S2. Relationship between ratio of integrated intensities of bending mode of water, $I_{\delta(HOH)}$, to that of C=O stretching mode, $I_{\nu(C=O)}$, ($I_{\delta(HOH)}/ I_{\nu(C=O)}$) and the ratio of weights of water, W_{water} to weight of CMCF, W_{CMCF}, (W_{water}/W_{CMCF}). The dotted line shows the calibration line used to estimate the water content of hydrogel.

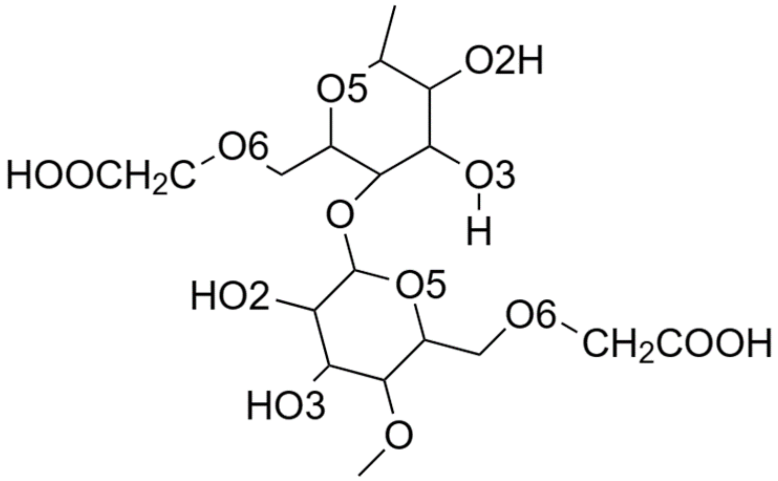

Figure S3. Classification of oxygen sites in CMC.

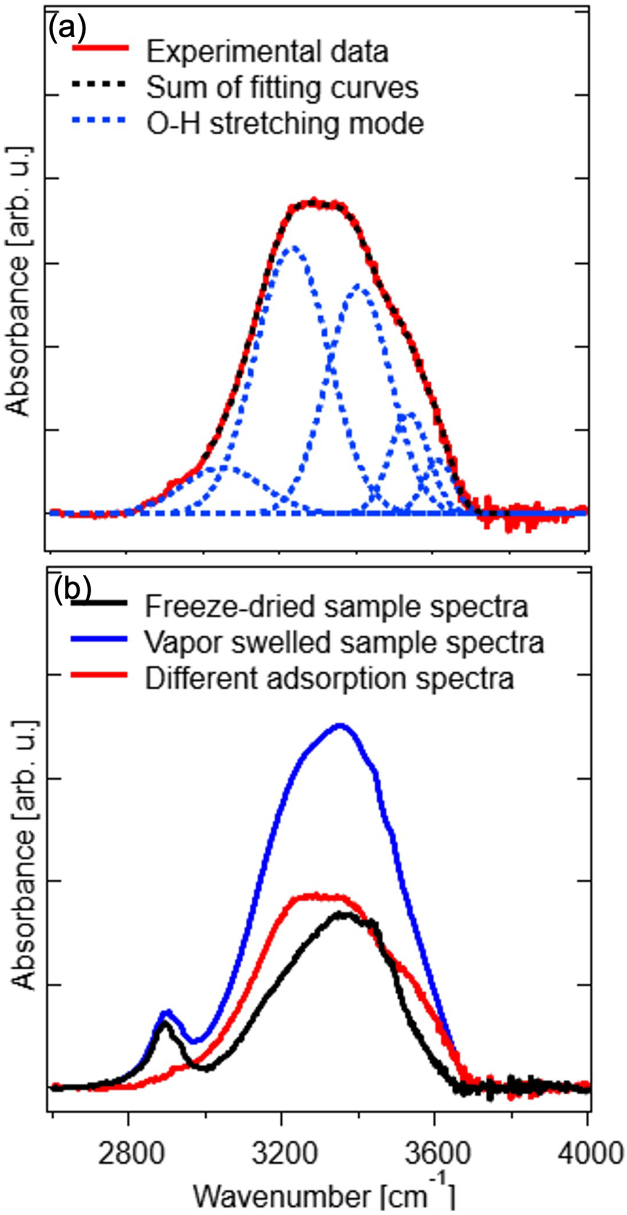

Figure S4. (a) Decomposed spectra of the O–H stretching modes of the CMCF hydrogel with 36.6 wt% in water content after the difference analysis. Red solid line represents the experimental spectrum. Blue and black dotted lines represent the fitting curves of the O–H stretching modes and sum of the fitted curves, respectively. The spectrum was analyzed using the difference absorption spectra to remove the O–H stretching peaks assigned to CMC. The spectrum of the freeze-dried sample (black line in (b)) was used as the basis to obtain the difference absorption spectra.

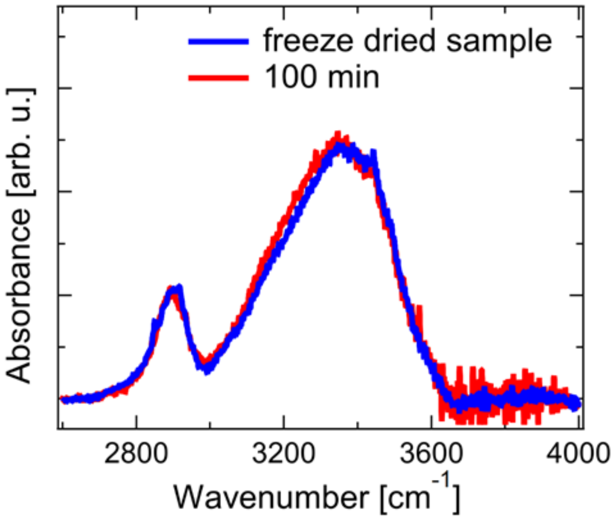

Figure S5. IR spectra of CMCF hydrogels of freeze-dried (red line) and natural dried for 100 min after vapor swelling (blue line).
